# Associations of Lipoprotein(a) With Coronary Atherosclerotic Burden and All-Cause Mortality in Patients With ST-Segment Elevation Myocardial Infarction Treated With Primary Percutaneous Coronary Intervention

**DOI:** 10.3389/fcvm.2021.638679

**Published:** 2021-06-15

**Authors:** Yuzhou Xue, Shen Jian, Wei Zhou, Qi Zhou, Jing Xiang, Yuansong Zhu, Zhenxian Xiang, Haonan Yang, Gang Liu, Suxin Luo

**Affiliations:** Department of Cardiology, The First Affiliated Hospital of Chongqing Medical University, Chongqing, China

**Keywords:** Coronary atherosclerotic burden, Lipoprotein(a), ST-segment elevation myocardial infarction, Gensini score, no-reflow, prognosis

## Abstract

**Background:** The coronary atherosclerotic burden in patients with ST-segment elevation myocardial infarction (STEMI) has been identified as the main predictor of prognosis. However, the association of lipoprotein(a) [Lp(a)], a well-established proatherogenic factor, with atherosclerotic burden in patients with STEMI is unclear.

**Methods:** In total, 1,359 patients who underwent percutaneous coronary intervention (PCI) for STEMI were included in analyses. Three prespecified models with adjustment for demographic parameters and risk factors were evaluated. Generalized additive models and restricted cubic spline analyses were used to assess the relationships of Lp(a) with Gensini scores and the no-reflow phenomenon. Kaplan–Meier curves were generated to explore the predictive value of Lp(a) for long-term all-cause mortality. Furthermore, mRNA expression levels of *LPA* in different groups were compared using the GEO database.

**Results:** Patients in the highest tertile according to Lp(a) levels had an increased incidence of heart failure during hospitalization. Furthermore, patients with high levels of Lp(a) (>19.1 mg/dL) had sharply increased risks for a higher Gensini score (*P*_for trend_ = 0.03) and no-reflow (*P*_for trend_ = 0.002) after adjustment for demographic parameters and risk factors. During a median follow-up of 930 days, 132 deaths (9.95%) were registered. Patients with high levels of Lp(a) (>19.1 mg/dL) had the worst long-term prognosis (*P*_for trend_ < 0.0001). In a subgroup analysis, patients with higher Lp(a) still had the highest all-cause mortality. Additionally, the mRNA expression levels of *LPA* in patients with STEMI with lower cardiac function were higher than those in other groups (*P* = 0.003). A higher coronary atherosclerotic burden was correlated with higher *LPA* expression (*P* = 0.01).

**Conclusion:** This study provides the first evidence that Lp(a) (at both the protein and mRNA levels) is independently associated with coronary atherosclerotic lesions and prognosis in patients with STEMI treated with PCI.

**Clinical Trial Registration:** http://www.chictr.org.cn/index.aspx, identifier: ChiCTR1900028516.

## Introduction

ST segment elevation myocardial infarction (STEMI) is still the main cause of morbidity and mortality worldwide, despite great progress in the development of treatments, especially mechanical infarct-related artery recanalization therapies (1, 2). Transmural STEMI and complications related to late or ineffective percutaneous coronary intervention (PCI) contribute to a worse prognosis (3, 4). These poor outcomes, at least in part, are related to the no-reflow phenomenon and non-infarct-related coronary atherosclerotic plaque burden (as evaluated by the Gensini score) (5, 6).

Lipoprotein(a) [Lp(a)], consisting of apolipoprotein(a) bound to apolipoprotein B-100 by a disulfide bridge, shows a similar structure to that of LDL-cholesterol (LDL-C) and high heterogeneity across ethnicities (7, 8). It has a proatherogenic component and is associated with atherosclerotic vascular diseases, including coronary artery disease (CAD), stroke, and peripheral artery disease (9). However, the relationship between Lp(a) and the coronary atherosclerotic burden in patients with STEMI undergoing PCI has not been investigated. The Gensini score, widely used for risk stratification in CAD patients, is a well-recognized predictor of atherosclerotic burden (10). Furthermore, recent studies have revealed that the no-reflow phenomenon after stenting is directly related to greater volume of cholesterol crystals, a larger necrotic core, more thin-cap fibroatheromas, and severe atherosclerotic burden in patients with infarct-related artery (11, 12). Hence, the aim of this study was to assess the relationships between (1) Lp(a) level and Gensini score and (2) Lp(a) level and the no-reflow phenomenon after infarct-related artery PCI, as well as clinical outcomes during follow-up.

## Materials and Methods

### Study Population

In total, 1,419 consecutive patients who underwent primary PCI for STEMI within 24 h after the onset of symptoms admitted to the First Affiliated Hospital of Chongqing Medical University were recruited in this prospective single-center study between January 2015 and December 2018. The diagnosis of STEMI was based on the presence of acute chest pain, new persistent ST-T elevations, or new left bundle-branch block in the electrocardiogram, and elevation of cardiac biomarkers, including creatine kinase-MB (CK-MB) and troponin I, above the 99th percentile upper reference limit (13). Exclusion criteria were as follows: lost to follow-up (*n* = 47) and myocardial infarction due to stent thrombosis (*n* = 13). Finally, 1,359 participants were included in the analysis.

The study was performed in accordance with the Declaration of Helsinki and was approved by the First Affiliated Hospital of Chongqing Medical University Ethics Committee. Every patient involved in the study provided written informed consent. This study has been registered in the Chinese Clinical Trial registry (URL: http://www.chictr.org.cn), with the unique identifier ChiCTR1900028516.

### Data Collection

Data pertaining to demographic profiles, medical history, comorbidities, clinical characteristics, laboratory parameters, medications, and echocardiographic and angiographic measurements were obtained from hospital records. Dyslipidemia was defined as low-density lipoprotein cholesterol (LDL-C) ≥3.36 mmol/L (130 mg/dL), high-density lipoprotein cholesterol (HDL-C) <1.03 mmol/L (40 mg/dL) for men or <1.29 mmol/L (50 mg/dL) for women, triglycerides ≥1.69 mmol/L (150 mg/dL), or the use of lipid-lowering medication (14). Quantification of the complexity of coronary atherosclerosis was determined by the Gensini score before PCI, which accurately reflects the atherosclerotic plaque burden (10, 15). A non-linear score was assigned to each lesion based on the severity and location of stenosis, and the sum of the lesion scores was defined as the Gensini score. The no-reflow phenomenon was defined as post-procedural thrombolysis in myocardial infarction (TIMI) flow grade of 0–II or TIMI flow grade 3 with a TIMI myocardial blush grade <2, persisting at the end of the PCI procedure (16). The diagnosis of heart failure required Killip class ≥II. Multivessel disease was recognized as at least 2 main branches of epicardial coronary artery with ≥70% stenosis or ≥50% stenotic lesions in the left main coronary artery (17).

Lipid profiles, including HDL-C, LDL-C, triglycerides, total cholesterol, and Lp(a), were measured in overnight fasting blood samples obtained within 24 h after PCI. Serum Lp(a) levels were calculated by an immunoturbidometric assay (Olympus, Beckman Coulter Instruments, Brea, CA, USA). Left ventricular ejection fraction (LVEF) was evaluated by echocardiography 24–48 h after admission.

### Follow Up

All patients enrolled in the study underwent regular follow-ups (typically every 3 months) by telephone reviews and office visits. Cardiac rehospitalization was defined as patients who were admitted to hospital due to cardiac events, including re-myocardial infarction, heart failure, cardiogenic shock, arrhythmia, major bleeding, and cardiac mortality. At the median follow-up of 930 days (interquartile range: 579–1,243 days), 132 deaths (9.95%) were recorded. The primary outcome was all-cause mortality. The end of follow-up was February 1, 2020.

### LPA Expression

Two published gene expression profiles (GSE59867 and GSE90074) from the Gene Expression Omnibus (GEO) database were used to evaluate the expression of *LPA*, which encodes Lp(a), in different groups. First, the expression levels of *LPA* in peripheral blood samples from patients with STEMI at admission (*n* = 111) were compared with those in samples from patients with stable CAD (*n* = 46) in GSE59867 (18). Furthermore, samples from patients with STEMI were stratified into four equal groups according to the plasma N-terminal pro-brain natriuretic peptide (NT-proBNP) level and LVEF (measured 6 months after STEMI). Among patients in the first and fourth quartiles, those with high levels of NT-proBNP and low LVEF (*n* = 9) were defined as the low cardiac function (CF) group, and patients with low levels of NT-proBNP and high LVEF (*n* = 8) were assigned to the high CF group. Other patients with STEMI (*n* = 94) were assigned to the intermediate CF group. Then, the differences in *LPA* expression among low, intermediate, and high CF as well as stable CAD groups were explored.

After excluding peripheral blood samples from patients with CAD class 0 (*n* = 18), 125 blood samples from GSE90074 were analyzed. The quantification of CAD classes was based on previously described criteria (19). The association between LPA expression and CAD class was also evaluated.

### Statistical Analysis

Baseline characteristics, angiographic profiles, and clinical outcomes are presented as means ± SD or medians (interquartile range) for continuous variables following a normal or non-normal distribution, respectively. Proportions for categorical variables were determined across the tertiles of Lp(a) level. Continuous variables were examined by ANOVA or Kruskal–Wallis tests for normally distributed and non-normally distributed variables, respectively. Categorical variables were examined by Pearson chi-squared tests, and Fisher's exact test was used for expected cell sizes of <5. The association between Lp(a) and the no-reflow phenomenon was investigated by a binary logistic regression analysis. Moreover, the association between Lp(a) and Gensini scores was assessed by a multivariate linear regression. Three prespecified models, including traditional risk factors for coronary atherosclerotic burden, were as follows. Model 1 (demographics) adjusted for age and gender. Model 2 adjusted for model 1 variables + hypertension, dyslipidemia, smoking, diabetes mellitus, and chronic kidney disease. Model 3 incorporated model 2 + symptom onset to balloon, body mass index, systolic blood pressure, hemoglobinA1c, triglycerides, total cholesterol, HDL-C, LDL-C, CK-MB, creatinine, high sensitivity C reactive protein (hsCRP), LVEF, prehospital thrombolysis, and lipid-lowering medication. Kaplan–Meier curves were generated to estimate the survival time with log-rank tests. Generalized additive models with the integrated smoothness estimation method were used to assess the relationship between Lp(a) and Gensini score in model 3 using the “mgcv” package. Moreover, a restricted cubic spline analysis was used to further explore the association between Lp(a) concentrations and risk for no-reflow after adjusting for model 3. All statistical analyses and visualization were performed using R (version 3.6.2). A 2-sided *P*-value of <0.05 was considered statistically significant.

## Results

### Baseline Characteristics

The average age was 63.4 ± 12.6 years, and 279 of 1,359 patients (20.5%) were female. Baseline characteristics stratified by Lp(a) levels are shown in Table 1. Older age, female, non-smoker, and heavy were associated with increased Lp(a) levels. Compared with the lowest tertile, subjects in the high tertile of Lp(a) were more likely to have higher levels of platelets, total cholesterol, LDL-C, HDL-C, and hsCRP and lower levels of hemoglobin and triglycerides. Additionally, increased rates of clopidogrel, anticoagulation, and diuretics and reduced rates of aspirin and ticagrelor administration were detected for the higher tertiles of Lp(a). However, use of prehospital thrombolysis and glycoprotein IIb/IIIa inhibitors did not differ significantly among patients in Lp(a) tertiles.

**Table 1:** Baseline characteristics by tertiles of lipoprotein(a) level.

**Variables**	**Lipoprotein(a) tertile (mg/dl)**			***P*-value**
	**<6.5 (*n =* 453)**	**6.5–19.1 (*n =* 452)**	**>19.1 (*n =* 454)**	
Age (years)	62.2 ± 12.6	63.8 ± 12.2	64.3 ± 12.8	0.04
Gender, n (%)				0.02
Female	74 (16.3%)	97 (21.4%)	108 (23.8%)	
Male	379 (83.7%)	356 (78.6%)	345 (76.2%)	
Smoking, n (%)	326 (72.0%)	307 (67.8%)	291 (64.2%)	0.04
Previous myocardial infarction, n (%)	21 (4.6%)	15 (3.3%)	20 (4.4%)	0.58
Hypertension, n (%)	230 (50.8%)	234 (51.7%)	238 (52.5%)	0.87
Dyslipidemia, n (%)	54 (11.9%)	39 (8.6%)	47 (10.4%)	0.27
Family history for coronary artery disease, n (%)	19 (4.2%)	25 (5.5%)	18 (4.0%)	0.48
Diabetes mellitus, n (%)	110 (24.3%)	94 (20.8%)	86 (19.0%)	0.14
Chronic kidney disease, n (%)	10 (2.2%)	14 (3.1%)	16 (3.5%)	0.53
Pulse (bpm)	82.1 ± 17.4	80.8 ± 18.3	83.0 ± 19.7	0.21
Systolic blood pressure (mmHg)	125.5 ± 24.8	123.5 ± 25.2	126.2 ± 25.7	0.26
Body mass index (Kg/m^2^)	24.6 ± 3.5	24.0 ± 3.7	23.5 ± 3.6	<0.001
White blood cell (10^9^/L)	13.8 ± 4.9	14.4 ± 6.5	11.6 ± 3.9	0.64
Hemoglobin (g/L)	140.2 ± 21.1	137.5 ± 19.1	136.0 ± 20.1	0.007
Platelet (10^9^/L)	203.0 ± 71.6	199.5 ± 58.5	210.6 ± 69.7	0.04
Hemoglobinba1c (%)	6.7 ± 1.6	6.6 ± 1.5	6.6 ± 1.8	0.60
Creatinine (umol/L)	73 (64–90)	76 (63–92)	77 (64–93)	0.31
Free triiodothyronine (pmol/L)	2.96 ± 0.72	2.89 ± 0.83	2.87 ± 0.67	0.16
Total cholesterol (mmol/L)	4.3 ± 1.1	4.4 ± 1.0	4.7 ± 1.1	<0.001
Triglycerides (mmol/L)	1.57 (1.02–2.47)	1.34 (0.94–1.98)	1.39 (1.04–1.96)	0.002
Low density lipoprotein cholesterol (mmol/L)	2.7 ± 0.9	2.8 ± 0.9	3.1 ± 1.0	<0.001
High-sensitivity C-reactive protein (mg/L)	4.47 (2.00–10.31)	6.17 (2.51–12.61)	5.70 (2.24–12.22)	0.006
Left ventricular end-diastolic dimension (mm)	49.4 ± 5.2	49.1 ± 5.5	49.4 ± 5.6	0.76
Left ventricle ejection fraction (%)	55.6 ± 7.1	55.1 ± 7.6	54.4 ± 8.0	0.08
Killip class on admission, n (%)				0.23
I	341 (75.3%)	340 (75.3%)	320 (70.4%)	
II	58 (12.8%)	48 (10.6%)	65 (14.3%)	
III	6 (1.3%)	14 (3.1%)	14 (3.1%)	
IV	48 (10.6%)	50 (11.0%)	55 (12.1%)	
Prehospital thrombolysis, n (%)	24 (5.3%)	38 (8.4%)	30 (6.6%)	0.18
Glycoprotein IIb/IIIa Inhibitors, n (%)	167 (36.9%)	169 (37.4%)	201 (44.3%)	0.06
Aspirin, n (%)	452 (99.8%)	448 (98.9%)	443 (97.8%)	0.02
Clopidogrel, n (%)	252 (55.6%)	274 (60.5%)	290 (64.0%)	0.04
Ticagrelor, n (%)	407 (89.8%)	378 (83.6%)	377 (83.0%)	0.04
Statin, n (%)	450 (99.3%)	452 (99.8%)	453 (100.0%)	0.33
β-blocker, n (%)	383 (84.5%)	375 (82.8%)	371 (81.9%)	0.56
Anticoagulation drug, n (%)	165 (37.1%)	164 (36.2%)	197 (43.5%)	0.02
Diuretics, n (%)	163 (36.0%)	188 (41.5%)	201 (44.4%)	0.03

### Clinical Outcomes and Multivariate Regression Analyses

**Figure 1:**
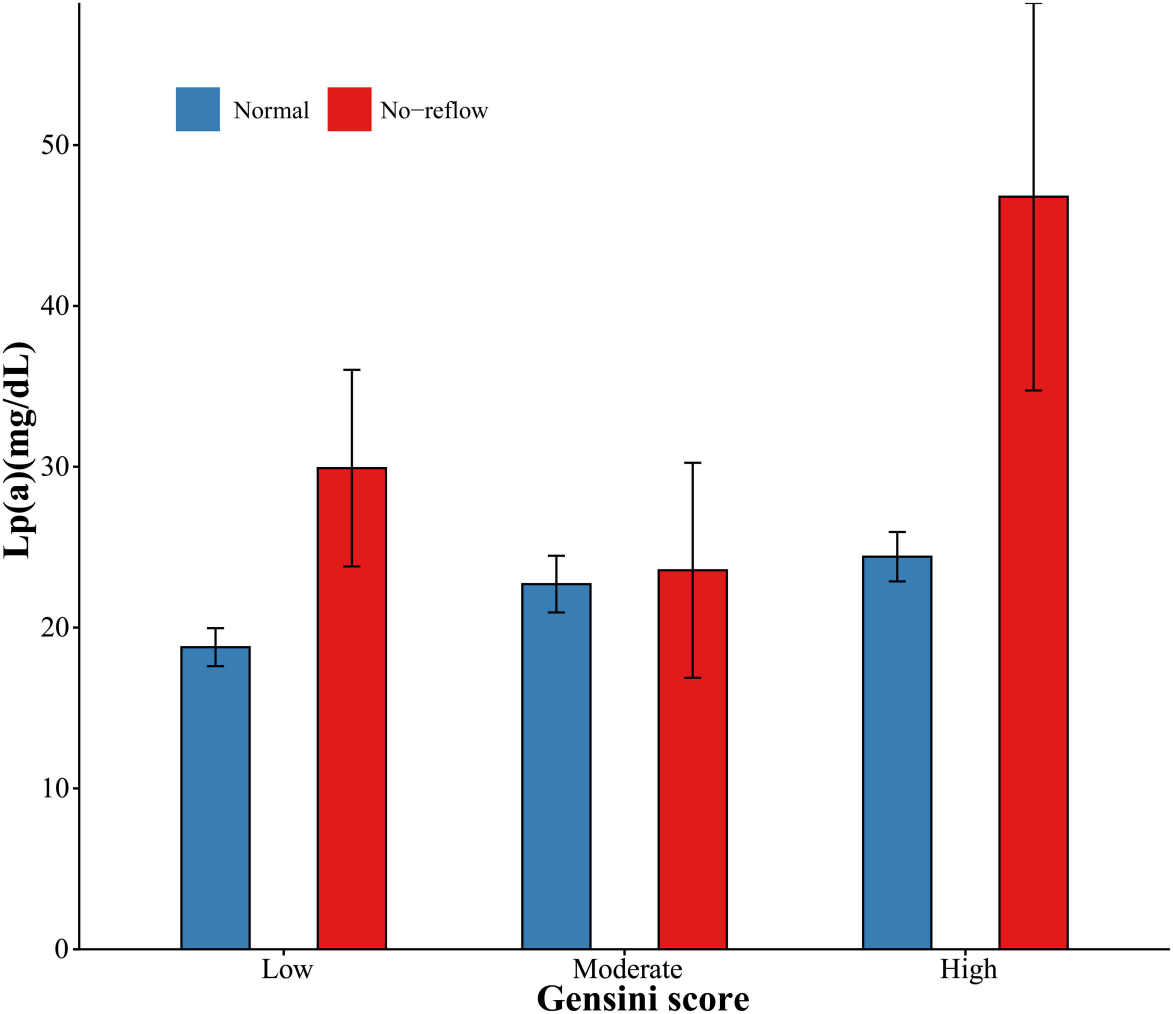
Levels of lipoprotein (a) with respect to Gensini scores and no-reflow.

Angiographic characteristics and clinical outcomes are described in Table 2. Acute kidney injury and heart failure during hospitalization were associated with high Lp(a) concentrations. Patients with no-reflow had increased Lp(a) concentrations. However, there were no differences in culprit artery, symptom onset to balloon, hospitalization duration, and death with respect to Lp(a) concentrations. Additionally, the levels of Lp(a) among categories of Gensini score and no-reflow are summarized in Figure 1. The patients with a high Gensini score and no-reflow had the highest levels of Lp(a).

**Table 2:** Angiographic characteristics and clinical outcomes during hospitalization by tertiles of lipoprotein(a) level.

**Variables**	**Lipoprotein(a) tertile (mg/dl)**			***P-*value**
	**<6.5 (*n =* 453)**	**6.5–19.1 (*n =* 452)**	**>19.1 (*n =* 454)**	
Culprit artery, n (%)				0.85
Left main artery	3 (0.7%)	3 (0.7%)	2 (0.4%)	
Left anterior descending artery	220 (48.5%)	235 (52.0%)	220 (48.3%)	
Left circumflex artery	47 (10.4%)	39 (8.6%)	46 (10.2%)	
Right coronary artery	169 (37.3%)	165 (36.5%)	171 (37.7%)	
Multivessel disease	14 (3.1%)	10 (2.2%)	15 (3.3%)	
Symptoms to balloon (h)	5 (3–9)	6 (3-11)	6 (4-11)	0.20
Gensini score	53.1 ± 26.0	53.6 ± 23.4	57.8 ± 25.4	<0.001
No-reflow phenomena, n (%)	21 (4.6%)	19 (4.2%)	41 (9.0%)	<0.001
Acute kidney injury, n (%)	2 (0.4%)	7 (1.5%)	10 (2.2%)	0.05
Heart failure, n (%)	87 (19.2%)	105 (23.2%)	126 (27.8%)	<0.01
Hospitalization duration (days)	8.2 ± 4.3	8.6 ± 4.7	8.9 ± 5.3	0.12
Death, n (%)	10 (2.2%)	8 (1.8%)	13 (2.9%)	0.57

**Figure 2:**
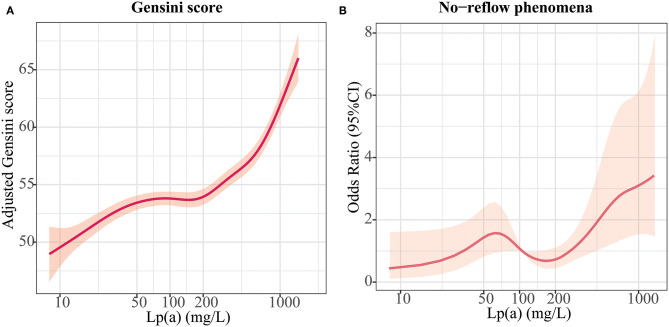
Relationships between lipoprotein (a) [Lp(a)] and atherosclerotic burden. **(A)** Association of Lp(a) with Gensini scores in an adjusted model; **(B)** Association of Lp(a) with no-reflow in an adjusted model.

Multivariate regression analyses were conducted to determine the correlations between Lp(a) and coronary atherosclerotic burden, as displayed in Table 3. The fully adjusted multivariate linear regression analysis revealed a strong positive relationship between Lp(a) and Gensini score (*P*_for trend_ = 0.03). After adjustment for cardiovascular risk factors, the odds ratios for no-reflow across Lp(a) tertiles (<6.5 mg/dL, 6.5–19.1 mg/dL, >19.1 mg/dL) were 0.36 (95%CI: 0.18–0.73), 0.42 (95%CI: 0.22–0.81), and 1 (referent), respectively (*P*_for trend_ 0.002). Figure 2 shows the generalized additive models of adjusted Gensini score across the level of Lp(a) and the restricted cubic spline of no-reflow risk across the increasing level of Lp(a). The risk of no-reflow tended to increase more sharply as Lp(a) levels increased.

**Table 3:** Multivariate analysis of the association between lipoprotein(a) and Gensini scores or no-reflow phenomenon.

	**Lipoprotein(a) tertile (mg/dL)**			
	**<6.5 (*n =* 453)**	**6.5–19.1 (*n =* 452)**	**>19.1 (*n =* 454)**	***P* for trend**
Gensini score, Beta (95%CI)				
Model 1	−4.2 (−7.5 to −1.0)	−4.3 (−7.5 to −1.1)	1.00 (Ref.)	
*P-*value	0.011	0.009	–	0.01
Model 2	−4.5 (−7.8 to −1.3)	−4.4 (−7.7 to −1.2)	1.00 (Ref.)	
*P-*value	0.006	0.007	–	0.006
Model 3	−3.8 (−7.1 to −0.4)	−3.7 (−7.0 to −0.35)	1.00 (Ref.)	
*P-*value	0.03	0.03	–	0.03
No-reflow, odds ratio (95% CI)				
Model 1	0.51 (0.29 to 0.88)	0.44 (0.25 to 0.78)	1.00 (Ref.)	
*P-*value	0.016	0.005	–	0.009
Model 2	0.50 (0.29 to 0.87)	0.44 (0.24 to 0.77)	1.00 (Ref.)	
*P-*value	0.014	<0.01	–	0.008
Model 3	0.36 (0.18 to 0.73)	0.42 (0.22 to 0.81)	1.00 (Ref.)	
*P-*value	0.005	0.010	–	0.002

*Model 1: adjusted by age and gender*.

*Model 2: model 1+hypertension, dyslipidemia, smoking, diabetes mellitus, chronic kidney disease*.

*Model 3: model 2+ symptom to balloon, body mass index, systolic blood pressure, hemoglobinA1c, triglycerides, total cholesterol, high density lipoprotein cholesterol, low density lipoprotein cholesterol, creatine kinase MB(CK-MB), creatinine, high sensitivity C reactive protein (hsCRP), left ventricular ejection fraction (LVEF), prehospital thrombolysis, lipid-lowering medication*.

### Lp(a) and Prognosis

**Figure 3:**
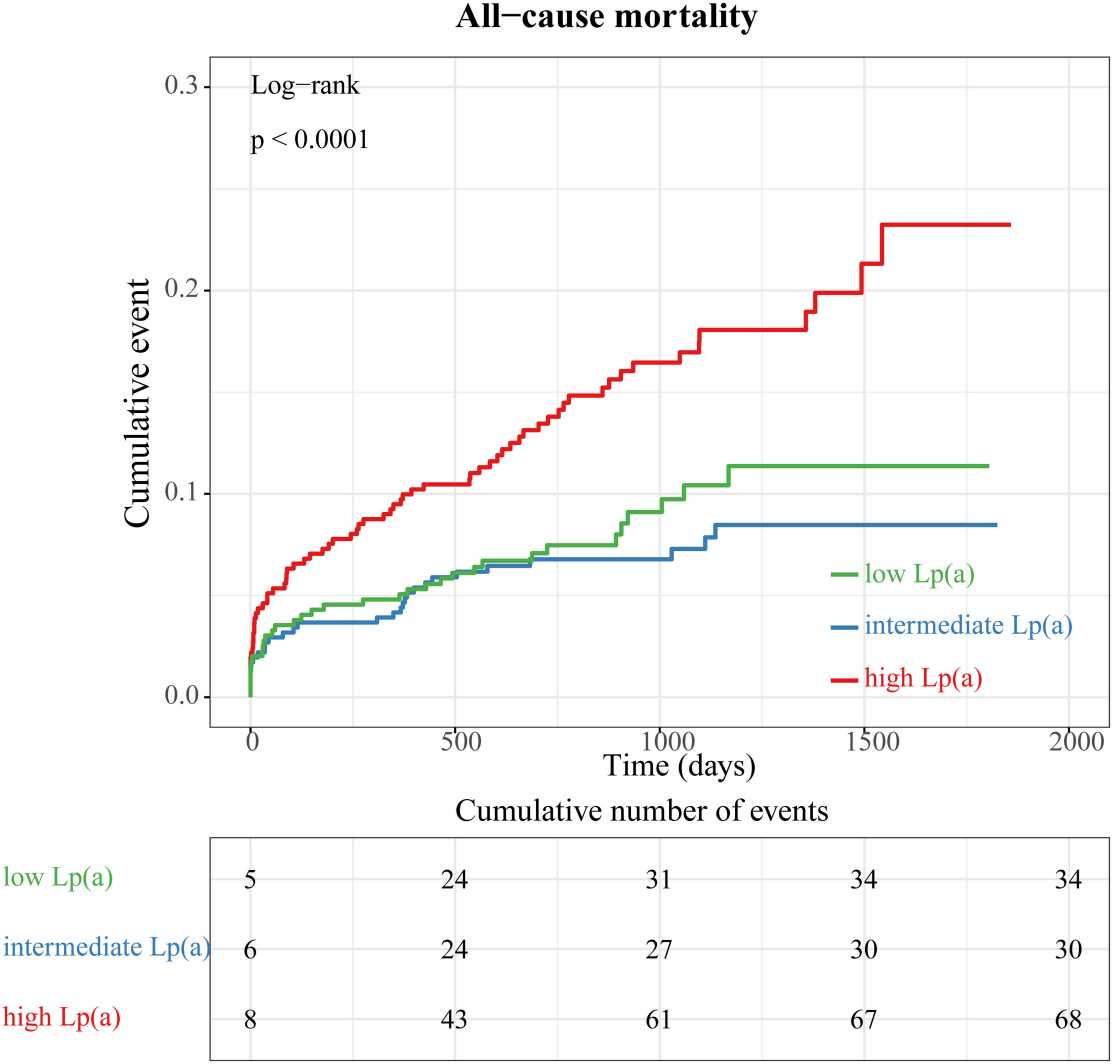
Kaplan–Meier survival curves according to tertiles of lipoprotein (a) concentrations (all-cause mortality).

**Figure 4:**
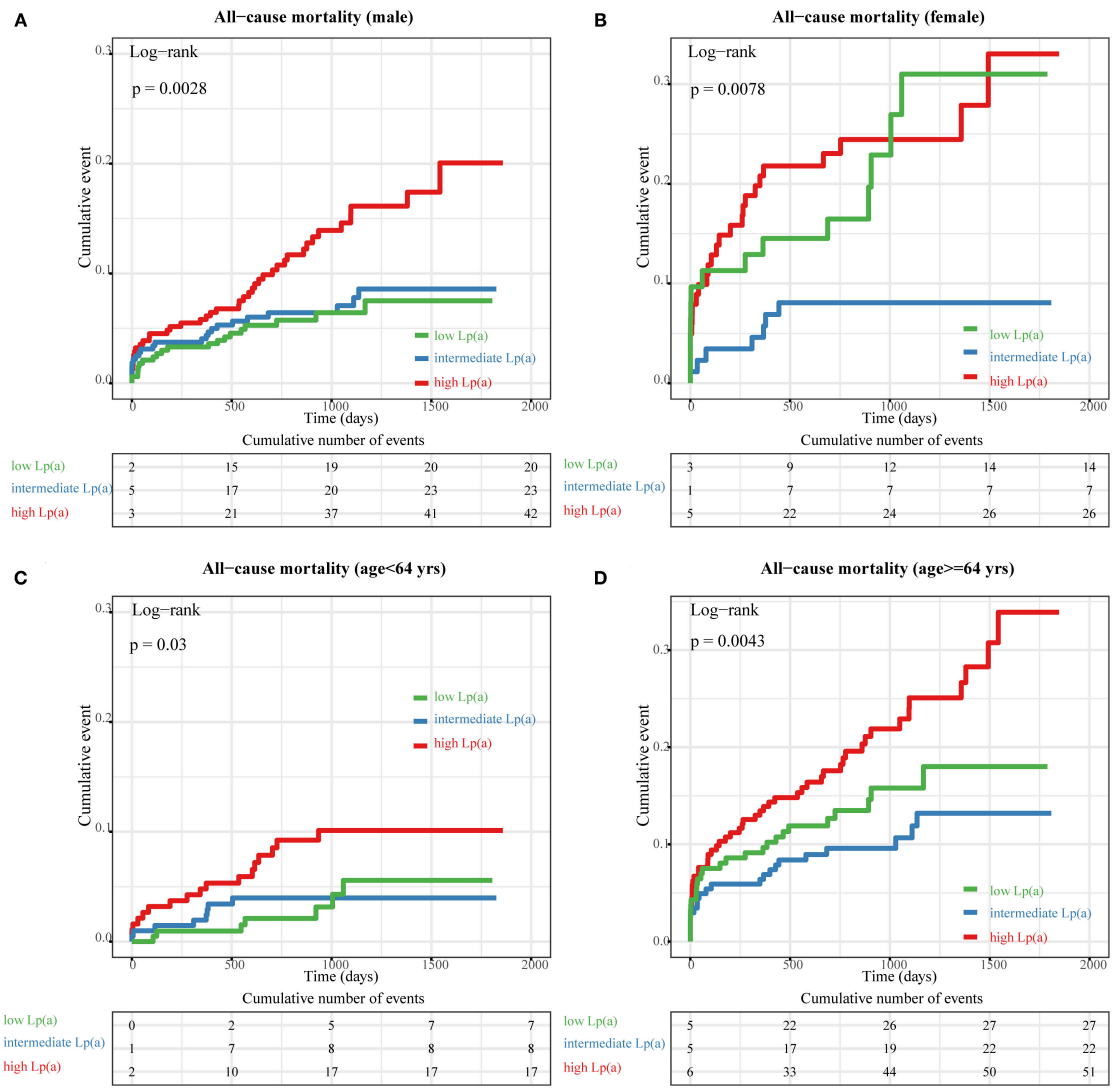
Subgroup analyses of Kaplan–Meier (KM) survival curves according to tertiles of lipoprotein (a) [Lp(a)]. The KM curves of all-cause mortality across different tertiles of Lp(a) in the following groups: **(A)** male, **(B)** female, **(C)** <64 years old, and **(D)** ≥64 years old.

When we compared all-cause mortality in patients with STEMI among categories of Lp(a) (Figure 3), we observed a significantly higher mortality in patients with Lp(a) >19.1 mg/dL than in those with Lp(a) <19.1 mg/dL (*P*_for trend_ < 0.0001). However, there was no significant difference between the low Lp(a) group (Lp < 6.5 mg/dL) and intermediate Lp(a) group (6.5 mg/dL to 19.1 mg/dL) by a log-rank test (*P* = 0.32). Furthermore, stratified analyses were applied to assess the correlation between Lp(a) and long-term prognosis with respect to gender and age (Figure 4). Regardless of gender or age, patients with high Lp(a) (>19.1 mg/dL) had the worst outcomes during follow-up.

Furthermore, we compared re-hospitalization events during follow-up among tertiles of Lp(a). In total, 405 (29.8%) all-cause and 365 (26.9%) cardiac re-hospitalization events were recorded in our database. We observed that patients with the highest tertile of Lp(a) had higher probabilities of both all-cause (*P*_for trend_ = 0.003) and cardiac (*P*_for trend_ = 0.0033) rehospitalization, as shown in Supplementary Figures 1, 2.

A multivariate Cox analysis was conducted to further identify the association of Lp(a) with low all-cause mortality, as shown in Supplementary Table 1. After adjusting for potential confounders, the high Lp(a) group was related to a worse prognosis during follow-up. The hazard ratios for all-cause mortality across Lp(a) tertiles were 0.59 (0.35–0.98), 0.47 (0.27–0.82), and 1 (referent), respectively (*P*_for trend_ = 0.03).

### *LPA* Expression and Coronary Atherosclerotic Burden

**Figure 5:**
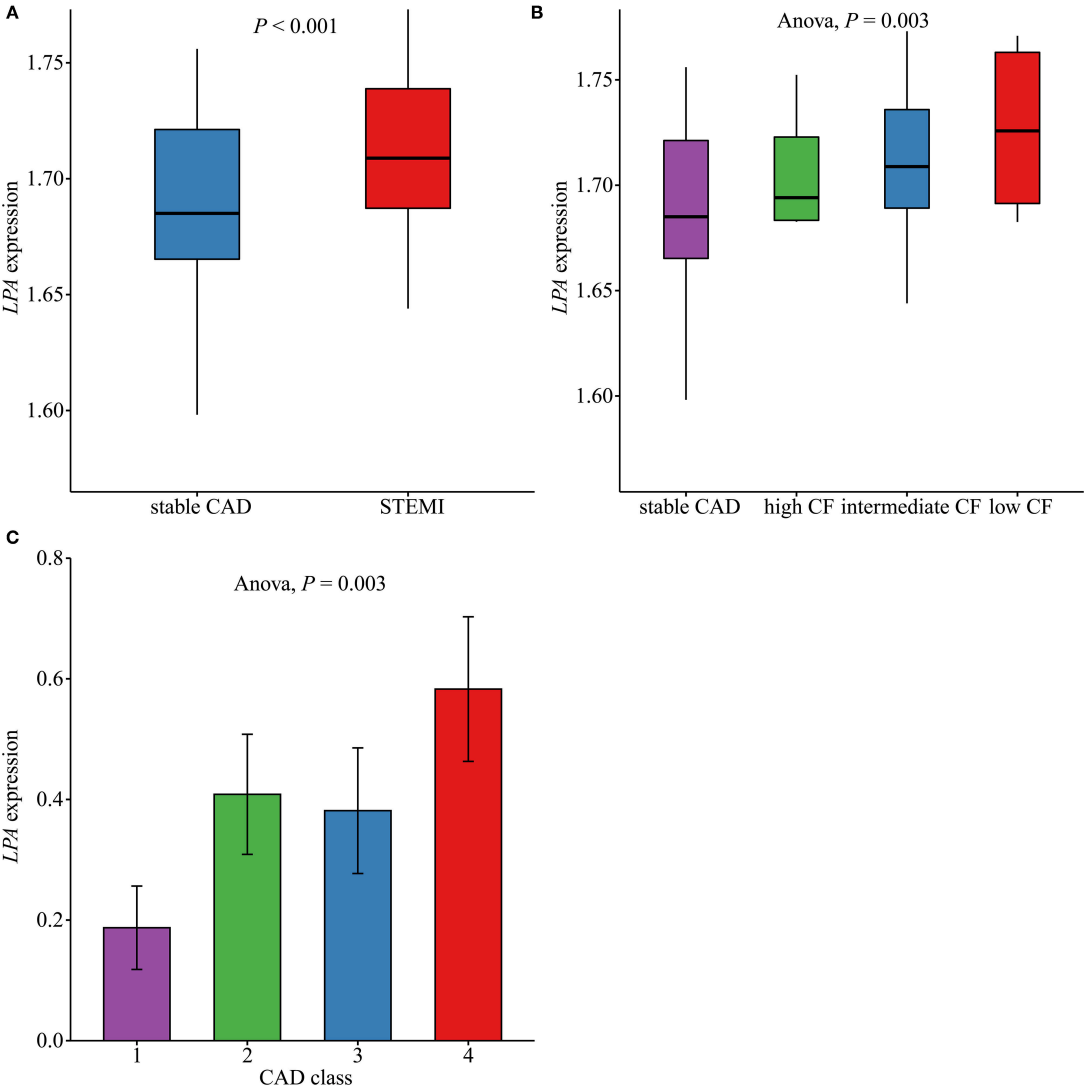
Comparison of LPA expression levels in different groups. **(A)** The LPA expression levels in ST-segment elevation myocardial infarction (STEMI, *n* = 111) and stable coronary artery disease (CAD, *n* = 46) in GSE59867 (*P* < 0.001). **(B)** LPA expression levels across low (*n* = 9), intermediate (*n* = 94), and high (*n* = 8) cardiac function (CF) and stable CAD (*n* = 46) groups in GSE59867 (*P* = 0.003). **(C)** LPA expression levels across CAD class = 1 (*n* = 32), 2 (*n* = 31), 3 (*n* = 26), and 4 (*n* = 36) in GSE90074.

The levels of *LPA* in different groups are shown in Figure 5. Compared with stable CAD, patients with STEMI had higher expression levels of *LPA* (*P* < 0.001) (Figure 5A). Furthermore, we observed a continuous decrease in *LPA* expression across low CF, intermediate CF, high CF, and stable CAD groups (*P*_for trend_ = 0.003) (Figure 5B). Moreover, the *LPA* expression levels for different CAD classes were compared, as summarized in Figure 5C. Patients with the most severe atherosclerotic burden (CAD class = 4) had the highest levels of *LPA* (*P* = 0.01).

## Discussion

In the current study, extremely high Gensini scores and the occurrence of no-reflow were observed in the 3rd tertile group for Lp(a) (>19.1 mg/dL). Furthermore, mRNA expression levels of Lp(a) were also correlated with the atherosclerotic burden and cardiac function. After adjustment for demographic and risk factors, high Lp(a) was still associated with worse all-cause mortality in a Cox analysis.

Many cohort studies have identified Lp(a) as a marker of cardiovascular risk, with an increase in cardiovascular disease events, even after statin treatment (20, 21). A study has shown that the Chinese population has the lowest concentrations and largest isoform sizes of Lp(a) among seven ethnics groups, with a relatively lower risk of myocardial infarction (22). The Gensini score, the most widely used angiographic scoring system, is significantly correlated with the average plaque burden and plaque area, as determined by intravascular ultrasound (10). Some studies have demonstrated a positive association of Lp(a) with Gensini scores in patients with stable CAD or in patients with type 2 diabetes mellitus (23, 24). In our study, high Lp(a) values were related to high Gensini scores, indicating that this relationship was preserved in patients with STEMI. Additionally, the association between Lp(a) and coronary atherosclerotic lesions still existed in the non-infarcted related artery, as shown in Supplementary Table 2. Animal experiments have shown that Lp(a) could bind to the vessel wall via glycosamino-glycans and migrate to the intimal space, which is the key process in atherogenesis (25). A pathway was recently proposed in which oxidized phospholipids bind with high affinity to Lp(a), which increases arterial wall cholesterol deposition, enhances foam cell formation, and induces monocyte-chemotactic activity in sub-endothelial spaces (26). As a result, high Lp(a) concentrations, at least in part, contribute to the severity of main epicardial atherosclerotic lesions.

Numerous studies have provided insight into the potential mechanisms underlying the no-reflow phenomenon in patients who underwent PCI for several decades. Coronary microvascular dysfunction can be sustained by ischemia and reperfusion injury and contributes to the development of no-reflow (4). Traditionally, the no-reflow phenomenon could be classified into structural and functional forms (27). In the functional type, the patency of distal microvessels is compromised by a loss of endothelium-mediated vasomotion, enhanced sympathetic activation, external microvasculature compression caused by edema myocytes, and accumulation of neutrophils and platelets. In the structural type, the irreversible obstruction of microvascular arteries is caused by necrotic myocytes, endothelial gaps, the release of oxygen free radicals, and distal embolization (3, 28, 29). Among these, plaque debris is one of the prominent pathophysiological causes for no reflow (30). Moreover, the presence of plaques with larger necrotic cores enhanced the risk of no reflow even after multivariable-adjusted analysis (11). In this study, we first found that high Lp(a) is strongly related to an increased risk of no-reflow, even after adjustment for traditional risk factors identified in previous studies (31, 32). We supposed that Lp(a) is associated with distal embolization and thus induces an increased probability of no-reflow. There are several explanations for this relationship. First, the thrombus volume as well as the presence of a lipid-rich plaque are related to the occurrence of coronary micro-embolization (33). The relationship between high Lp(a) and a larger coronary atherosclerotic plaque has been discussed previously. Second, a high level of Lp(a) resulting from a low number of *LPA* kringle-IV type 2 repeats could slow fibrinolysis and thus indirectly promote thrombosis, as kringle structures are homologous to plasminogen (34, 35). Overall, we predicted an association between Lp(a) and distal embolization in the no-reflow phenomenon.

Furthermore, little is known about the relationship between heart failure and Lp(a). Kamstrup et al. suggested that elevated Lp(a) levels are correlated with heart failure, consistent with a causal relationship in the general population (36). In the ARIC study, participants with increased Lp(a) had an increased risk of incident heart failure hospitalization; however, this association was no longer significant after adjusting (37). Furthermore, the Multi-Ethnic Study of Atherosclerosis revealed that a relationship between Lp(a) and heart failure with preserved ejection fraction (HFpEF) is only found in the general Caucasian population (38). As determined by multivariate logistic regression analyses (Supplementary Table 3), we observed that a gradually increased prevalence of heart failure during hospitalization appeared across tertiles of Lp(a) in our study. We found that higher *LPA* expression was also related to reduced cardiac function, consistent with the analyses of protein levels. Hence, Lp(a) could contribute to the pathophysiological process of heart failure. However, further research is needed to determine the precise mechanism underneath the association of heart failure with Lp(a).

Conflicting results have been obtained regarding the association between Lp(a) and risk for all-cause mortality in patients with acute coronary syndrome. Zhou et al. found that the hazard ratio for the incidence of subsequent cardiovascular events associated with one unit (mg/dL) increase of ln[Lp(a)] was 1.51 after adjustment (39). Three recent studies have shown that patients who underwent PCI or coronary artery bypass grafting with high Lp(a) had a worse mortality (40–42). Conversely, another study of 1,245 patients with acute coronary syndrome stratified into four groups according to the quartile of Lp(a) showed that Lp(a) is not associated with long-term mortality (43). In our study, high Lp(a) (>19.1 mg/dL) was strongly related to the long-term prognosis in the total population and in subgroup analyses, and a Cox regression analysis further revealed that Lp(a) is an indicator of all-cause mortality even after adjusting for risk factors. There are two potential explanations for this relationship. First, we only enrolled patients with STEMI treated with PCI, which probably minimized the effect of the heterogeneous population on long-term mortality in previous studies. Second, the JMS cohort study, including 10,413 individuals from the general population of Japan, found that low levels (<8 mg/dL) of Lp(a) resulted in a higher hazard ratio (1.43, 95%CI 1.21–1.68) for all-cause mortality (44); this finding concurred with the observation that Lp(a) is inversely related to major bleeding (45). In patients with STEMI treated with PCI, anti-platelet medication is required for at least 1 year, which could enhance the risk of major bleeding. Hence, this would result in a U-shaped association of Lp(a) with long-term prognosis, as lower values of Lp(a) would aggravate the risk of major bleeding, and higher levels would increase the coronary atherosclerotic burden.

In the stratification analysis, high Lp(a) levels was still associated with increased risk of death for males and, females, and for patients older and younger than 64 years. Furthermore, female patients with low Lp(a) (<6.5 mg/dL) levels had significantly enhanced all-cause mortality compared with those with intermediate Lp(a) (6.5–19.1 mg/dL) levels (log-rank test: *P* = 0.028). Long-term mortality did not differ significantly for patients between the low and intermediate Lp(a) tertiles in other subgroups. Therefore, the baseline characteristics for female patients were compared between the low and intermediate Lp(a) tertiles, as shown in Supplementary Table 4. Most variables were comparable between the low and intermediate Lp(a) tertiles except for hsCRP. However, a higher in-hospital mortality was observed for female patients in the low Lp(a) tertile (*P* = 0.04). The potential reason for the differences in hospitalization and long-term mortality may be that the average age of female patients with low Lp(a) levels was older than those with intermediate Lp(a) levels, although not significantly (*P* = 0.14). A larger study of female STEMI patients should be conducted in the future to validated these findings.

A recent meta-analysis revealed that statin therapy elevated *LPA* mRNA expression, resulting in a significant increase in plasma Lp(a) level (46). Moreover, emerging antisense oligonucleotides (IONIS-APO(a) RX and IONIS-APO(a)-L RX), which can specifically bind with *LPA* mRNA, have demonstrated the ability to effectively decrease Lp(a) concentrations (47). However, Lp(a)-lowering therapies, including antisense oligonucleotides, had not been implemented in our study. Furthermore, few studies have investigated Lp(a)-lowering therapy in STEMI patients (48). Our study is also the first to identify a positive relationship between *LPA* level and atherosclerotic burden and also cardiac function. As such, our findings could be helpful for further clinical application of specifical antisense oligonucleotides, including IONIS-APO(a) RX and IONIS-APO(a)-L RX, especially in STEMI patients.

Our study had several limitations. First, we used the Beckman assay to determine the values of Lp(a) in mg/dL, instead of nmol/L. As a result, we could not adjust for the effect of the number of *LPA* kringle-IV type 2 repeats. Second, although multivariate analyses were used to adjust for potential variables, residual confounding from unmeasured variables cannot be excluded. Third, atherosclerotic events, including another acute coronary syndrome, stroke, and cardiac death, during the long-term follow-up were not recorded in this study. Therefore, the relationship between Lp(a) level and atherosclerotic outcomes could not be elucidated. Furthermore, the Lp(a) value was only measured once for each STEMI patient; convalescent levels of Lp(a) during hospitalization and after discharge would be helpful to identify specific factors responsible for changes in Lp(a) and to explore the association between Lp(a) fluctuations and clinical outcomes.

In conclusion, we demonstrated that Lp(a) is independently associated with atherosclerotic burden in patients with STEMI treated with PCI. Furthermore, a high level of Lp(a) was a strong predictor of long-term mortality.

## Data Availability Statement

The raw data supporting the conclusions of this article will be made available by the authors, without undue reservation.

## Ethics Statement

The studies involving human participants were reviewed and approved by the First Affiliated Hospital of Chongqing Medical University Ethics Committee. The patients/participants provided their written informed consent to participate in this study.

## Author Contributions

YX and SL drafted the manuscript. SJ revised the manuscript critically. WZ, JX, and HY collected the data. YX, YZ, GL, and ZX analyzed the data. SL, YX, and QZ designed the study. SL revised the final version of the manuscript. All authors read and approved the final version of the manuscript.

## Conflict of Interest

The authors declare that the research was conducted in the absence of any commercial or financial relationships that could be construed as a potential conflict of interest.

## Supplementary Material

Supplementary Figure 1 Kaplan–Meier survival curves according to tertiles of lipoprotein(a) (all-cause rehospitalization).

Supplementary Figure 2 Kaplan–Meier survival curves according to tertiles of lipoprotein(a) (cardiac rehospitalization).

Supplementary Table 1 Cox analysis of the association between lipoprotein(a) and all-cause mortality.

Supplementary Table 2 Multivariate linear regression analyses of the association between lipoprotein(a) and Gensini scores for non-infarct related arteries in different models.

Supplementary Table 3 Multivariate analysis of the association between lipoprotein(a) and new-onset heart failure during hospitalization in different models.

Supplementary Table 4 Baseline characteristics between female patients with low and intermediate lipoprotein(a) levels.
