# Supplementary figures and images for: Associations of Lipoprotein(a) With Coronary Atherosclerotic Burden and All-Cause Mortality in Patients With ST-Segment Elevation Myocardial Infarction Treated With Primary Percutaneous Coronary Intervention
Source: Front Cardiovasc Med. 2021 Jun 15;8:638679. doi: 10.3389/fcvm.2021.638679 (PMC8239367; doi:10.3389/fcvm.2021.638679)

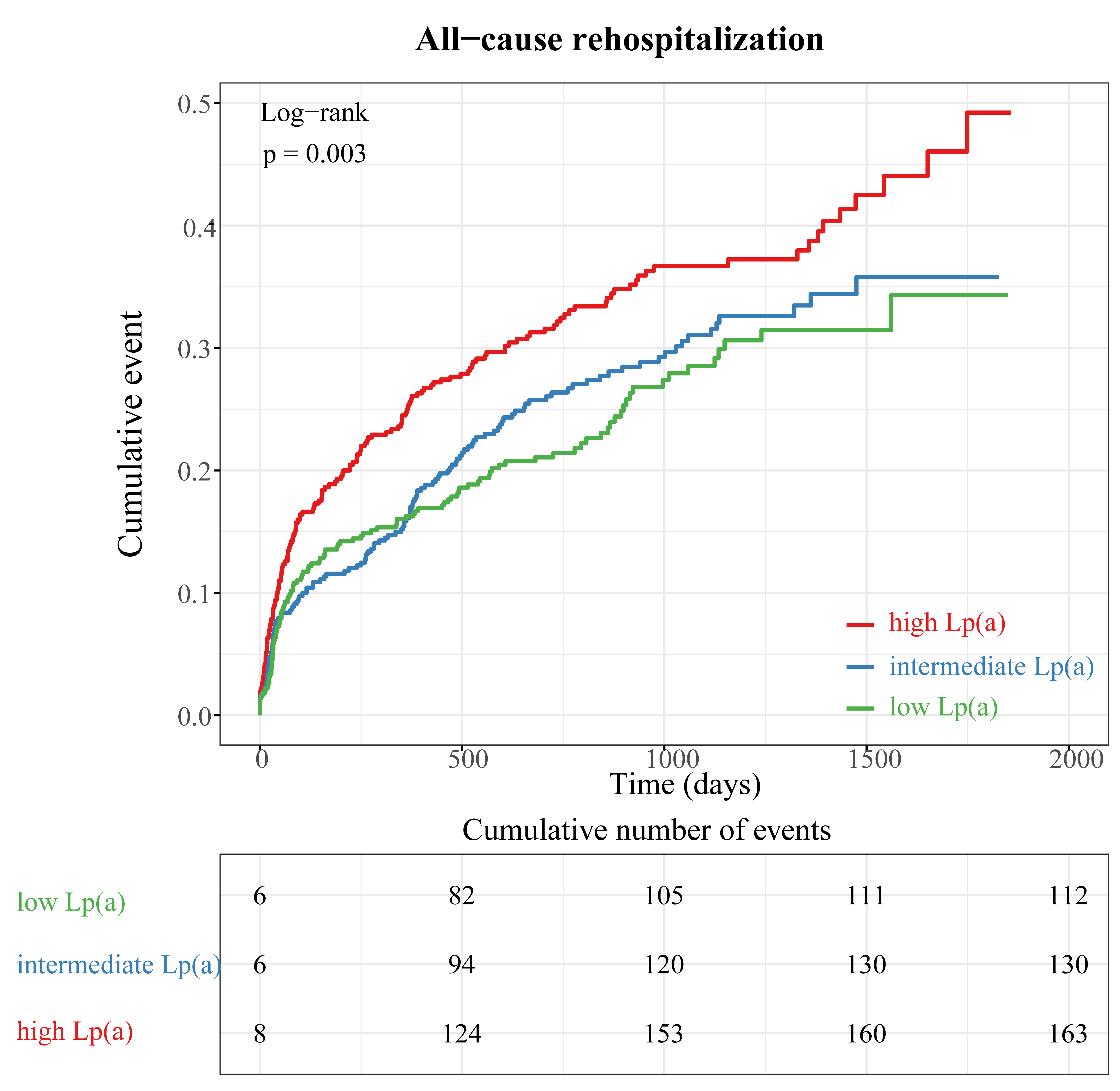

Supplement: Supplementary Figure 1 — Kaplan–Meier survival curves according to tertiles of lipoprotein(a) (all-cause rehospitalization). [file Image_1.TIF]

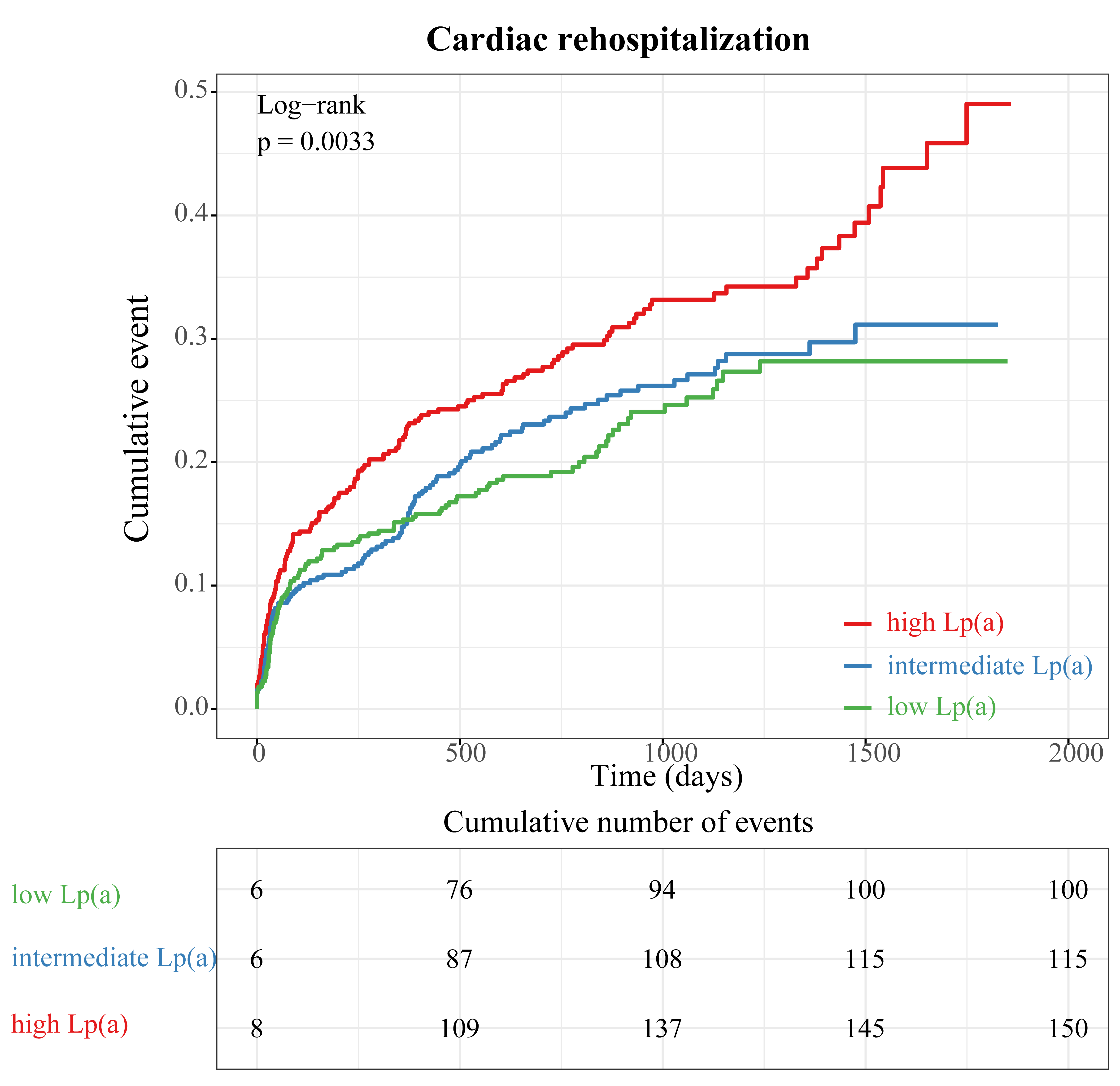

Supplement: Supplementary Figure 2 — Kaplan–Meier survival curves according to tertiles of lipoprotein(a) (cardiac rehospitalization). [file Image_2.TIF]
