# Supplementary material for: Associations of Lipoprotein(a) With Coronary Atherosclerotic Burden and All-Cause Mortality in Patients With ST-Segment Elevation Myocardial Infarction Treated With Primary Percutaneous Coronary Intervention
Source: Front Cardiovasc Med. 2021 Jun 15;8:638679. doi: 10.3389/fcvm.2021.638679 (PMC8239367; doi:10.3389/fcvm.2021.638679)
Supplement: Supplementary Table 1 — Cox analysis of the association between lipoprotein(a) and all-cause mortality. [file Table_1.docx]

Supplementary Table 1. Cox analysis of association between lipoprotein (a) and all-cause mortality.

|  | Lipoprotein(a) tertile (mg/dL) | | |  |
| --- | --- | --- | --- | --- |
|  | <6.5(n=453) | 6.5-19.1(n=452) | >19.1(n=454) | *P* for trend |
| Model 1, HR (95% CI) | 0.43 (0.27-0.69) | 0.40 (0.25-0.64) | 1.00 (Ref.) |  |
| P value | <0.001 | <0.001 | -- | <0.001 |
| Model 2, HR (95% CI) | 0.48 (0.30-0.76) | 0.41 (0.26-0.66) | 1.00 (Ref.) |  |
| P value | 0.002 | <0.001 | -- | <0.001 |
| Model 3, HR (95% CI) | 0.47 (0.29-0.75) | 0.40 (0.24-0.64) | 1.00 (Ref.) |  |
| P value | 0.002 | <0.001 | -- | 0.003 |
| Model 4, HR (95% CI) | 0.59 (0.35-0.98) | 0.47 (0.27-0.82) | 1.00 (Ref.) |  |
| P value | 0.04 | 0.007 | -- | 0.03 |

Model 1: unadjusted.

Model 2: adjusted by age and gender.

Model 3: model 2+hypertension, dyslipidemia, smoking, diabetes mellitus, chronic kidney disease.

Model 4: model 3+ symptom to balloon, body mass index, systolic blood pressure, hemoglobinA1c, triglycerides, total cholesterol, high density lipoprotein cholesterol, low density lipoprotein cholesterol, creatine kinase MB(CK-MB), creatinine, high sensitivity C reactive protein (hsCRP), left ventricular ejection fraction (LVEF), prehospital thrombolysis, lipid-lowering medication.
