# Supplementary material for: Associations of Lipoprotein(a) With Coronary Atherosclerotic Burden and All-Cause Mortality in Patients With ST-Segment Elevation Myocardial Infarction Treated With Primary Percutaneous Coronary Intervention
Source: Front Cardiovasc Med. 2021 Jun 15;8:638679. doi: 10.3389/fcvm.2021.638679 (PMC8239367; doi:10.3389/fcvm.2021.638679)
Supplement: Supplementary Table 2 — Multivariate linear regression analyses of the association between lipoprotein(a) and Gensini scores for non-infarct related arteries in different models. [file Table_2.docx]

Supplementary Table 2. Multivariable linear regression analyses of association between lipoprotein (a) and Gensini score of non-infarct related arteries in different models.

| Variable |  | Models | β value | 95%CI | P value |
| --- | --- | --- | --- | --- | --- |
| Ln[Lp(a)] | | Model 1 | 1.35 | (0.50 to 2.21) | 0.002 |
|  |  | Model 2 | 1.48 | (0.627 to 2.33) | 0.001 |
|  |  | Model 3 | 1.38 | (0.48 to 2.27) | 0.003 |
| Lipoprotein(a) tertile (mg/dl) |  | Model 3 |  |  |  |
|  | <6.5(n=439) |  | -3.51 | (-6.15 to -0.87) | 0.01 |
|  | 6.5-19.1(n=442) |  | -3.62 | (-6.25 to -1.01) | 0.007 |
|  | >19.1(n=439) |  | 1 (Ref.) |  | -- |

Excluding for the patients with multivessel coronary artery disease.

Model 1: adjusted by age and gender

Model 2: model 1+hypertension, dyslipidemia, smoking, diabetes mellitus, chronic kidney disease

Model 3: model 2+ symptom to balloon (h), body mass index, systolic blood pressure, hemoglobinA1c, triglycerides, total cholesterol, high density lipoprotein cholesterol, low density lipoprotein cholesterol, creatine kinase MB(CK-MB), creatinine, high sensitivity C reactive protein (hsCRP), left ventricular ejection fraction (LVEF), prehospital thrombolysis, lipid-lowering medication.
