# Supplementary material for: Associations of Lipoprotein(a) With Coronary Atherosclerotic Burden and All-Cause Mortality in Patients With ST-Segment Elevation Myocardial Infarction Treated With Primary Percutaneous Coronary Intervention
Source: Front Cardiovasc Med. 2021 Jun 15;8:638679. doi: 10.3389/fcvm.2021.638679 (PMC8239367; doi:10.3389/fcvm.2021.638679)
Supplement: Supplementary Table 3 — Multivariate analysis of the association between lipoprotein(a) and new-onset heart failure during hospitalization in different models. [file Table_3.docx]

Supplementary Table 3. Multivariable analysis of association between lipoprotein (a) and new onset heart failure during hospitalization in different models.

| Variable | Models | OR | 95%CI | P value |
| --- | --- | --- | --- | --- |
| Ln[Lp(a)] | Model 1 | 1.13 | 1.02-1.27 | 0.023 |
|  | Model 2 | 1.17 | 1.02-1.27 | 0.02 |
|  | Model 3 | 1.13 | 1.01-1.28 | 0.04 |
|  | Model 4 | 1.12 | 0.98-1.26 | 0.08 |
|  | Model 5 | 1.10 | 0.97-1.25 | 0.14 |
|  | Model 6 | 1.09 | 0.96-1.23 | 0.21 |
| No-reflow | Model 4 | 1.93 | 1.13-3.29 | 0.015 |
|  | Model 6 | 1.73 | 1.00-3.01 | 0.05 |
| Gensini score | Model 5 | 1.03 | 1.02-1.04 | <0.001 |
|  | Model 6 | 1.02 | 1.01-1.03 | <0.001 |

Model 1: adjusted by age and gender

Model 2: model 1+hypertension, dyslipidemia, smoking, diabetes mellitus, chronic kidney disease

Model 3: model 2+ symptom to balloon, body mass index, systolic blood pressure, hemoglobinA1c, triglycerides, total cholesterol, high density lipoprotein cholesterol, low density lipoprotein cholesterol, creatine kinase MB(CK-MB), creatinine, high sensitivity C reactive protein (hsCRP), left ventricular ejection fraction (LVEF), prehospital thrombolysis, lipid-lowering medication.

Model 4: model 3+ no-reflow.

Model 5: model 3+ Gensini score

Model 6: model 3+ no-reflow, Gensini score
