# Supplementary material for: Associations of Lipoprotein(a) With Coronary Atherosclerotic Burden and All-Cause Mortality in Patients With ST-Segment Elevation Myocardial Infarction Treated With Primary Percutaneous Coronary Intervention
Source: Front Cardiovasc Med. 2021 Jun 15;8:638679. doi: 10.3389/fcvm.2021.638679 (PMC8239367; doi:10.3389/fcvm.2021.638679)
Supplement: Supplementary Table 4 — Baseline characteristics between female patients with low and intermediate lipoprotein(a) levels. [file Table_4.docx]

Supplementary Table 4. Baseline characteristics between female patients with low Lp(a) and intermediate Lp(a).

| Variables | Lp(a) (mg/dL) | | *P* value |
| --- | --- | --- | --- |
|  | <6.5(n=74) | 6.5-19.1(n=97) |  |
| Age (years) | 71±10.9 | 68±11.5 | 0.14 |
| Smoking, n (%) | 5 (6.7%) | 8(8.2%) | 0.78 |
| Previous myocardial infarction, n (%) | 1(1.3%) | 5(5.1%) | 0.24 |
| Hypertension, n (%) | 46(62.2%) | 61(62.8%) | 1.00 |
| Dyslipidemia, n (%) | 8(10.8%) | 6(6.2%) | 0.4 |
| Family history for  coronary artery disease, n (%) | 3(4.1%) | 6(6.2%) | 0.73 |
| Diabetes mellitus, n (%) | 17(23.0%) | 30(30.9%) | 0.30 |
| Chronic kidney disease, n (%) | 2(2.7%) | 5(5.1%) | 0.70 |
| Pulse (bpm) | 80.0 (70.0-96.0) | 82.0(70.0-90.0) | 0.92 |
| Systolic blood pressure (mmHg) | 121.4±26.4 | 125.4±27.9 | 0.34 |
| Body mass index (Kg/m^2^) | 23.7±3.8 | 23.0±5.1 | 0.36 |
| White blood cell (10^9^/L) | 10.3(8.4-13.0) | 10.5(8.9-12.9) | 0.55 |
| Hemoglobin (g/L) | 121.7±16.0 | 125.7±15.8 | 0.10 |
| Platelet (10^9^/L) | 213.9±73.3 | 215.7±63.6 | 0.87 |
| hemoglobinbA1c (%) | 6.5±1.5 | 6.9±1.9 | 0.14 |
| Creatinine (umol/L) | 64.0(53.0-81.0) | 59.0(53.0-76.0) | 0.50 |
| Free triiodothyronine (pmol/L) | 3.0±1.4 | 2.6±0.5 | 0.06 |
| Total cholesterol (mmol/L) | 4.3±1.2 | 4.5±1.0 | 0.22 |
| Triglycerides (mmol/L) | 1.6(1.0-2.0) | 1.5(09-1.9) | 0.34 |
| Low density lipoprotein cholesterol (mmol/L) | 2.7±1.0 | 2.9±0.9 | 0.14 |
| high-sensitivity C-reactive protein (mg/L) | 5.1(2.0-12.5) | 8.8(4.0-17.1) | <0.01 |
| Left ventricular end-diastolic dimension (mm) | 46.2±4.3 | 46.5±4.5 | 0.70 |
| Left ventricle ejection fraction (%) | 56.0±6.8 | 54.7±8.2 | 0.31 |
| Killip class on admission, n (%) |  |  | 0.47 |
| I | 45(60.8%) | 69(71.1%) |  |
| II | 16(21.6%) | 13(13.4%) |  |
| III | 3(4.1%) | 4(4.1%) |  |
| IV | 10(13.5%) | 11(11.3%) |  |
| Aspirin, n (%) | 74(100%) | 93(95.9%) | 0.13 |
| Clopidogrel, n (%) | 39(53%) | 64(66%) | 0.09 |
| Ticagrelor, n (%) | 66(89.2%) | 80(82.5%) | 0.28 |
| Statin, n (%) | 72(97.2%) | 97(100%) | 0.19 |
| β-blocker, n (%) | 60(81.1%) | 85(87.6%) | 0.28 |
| Anticoagulation drug, n (%) | 6(8.1%) | 7(7.2%) | 0.41 |
| Diuretics, n (%) | 39(52.7%) | 51(52.5%) | 1.00 |
| Prehospital thrombolysis, n (%) | 1(1.4%) | 1(1.0%) | 1.00 |
| Culprit artery, n (%) |  |  | 0.15 |
| Left main artery | 1(1.4%) | 0(0%) |  |
| Left anterior descending artery | 32(43.2%) | 52(53.6%) |  |
| Left circumflex artery | 7(9.5%) | 13(13.4%) |  |
| Right coronary artery | 29(39.2%) | 30(30.9%) |  |
| Multivessel disease | 5(6.7%) | 2(2.1%) |  |
| Symptoms to balloon (h) | 7.0(5.0-11.0) | 7.0(4.0-11.0) | 0.95 |
| Gensini score | 54.2±27.4 | 56.7±21.3 | 0.49 |
| No-reflow phenomena, n (%) | 6(8.1%) | 7(7.2%) | 1.00 |
| Heart failure, n (%) | 28(37.8%) | 27(27.8%) | 0.19 |
| Hospitalization duration (days) | 8.1±5.0 | 10.3±6.5 | 0.02 |
| Death, n (%) | 6(8.1%) | 1(1.0%) | 0.04 |
